# Homologous overexpression of NpDps2 and NpDps5 increases the tolerance for oxidative stress in the multicellular cyanobacterium *Nostoc punctiforme*

**DOI:** 10.1093/femsle/fny198

**Published:** 2018-08-13

**Authors:** Xin Li, Henna Mustila, Ann Magnuson, Karin Stensjö

**Affiliations:** Department of Chemistry-Ångström Laboratory, Uppsala University, Box 523, SE 75120 Uppsala, Swedens

**Keywords:** Dps protein, cyanobacteria, *Nostoc*, ferritin, iron, photosynthesis, hydrogen peroxide, light-stress, ROS

## Abstract

The filamentous cyanobacterium *Nostoc punctiforme* has several oxidative stress-managing systems, including Dps proteins. Dps proteins belong to the ferritin superfamily and are involved in abiotic stress management in prokaryotes. Previously, we found that one of the five Dps proteins in *N. punctiforme*, NpDps2, was critical for H_2_O_2_ tolerance. Stress induced by high light intensities is aggravated in *N. punctiforme* strains deficient of either NpDps2, or the bacterioferritin-like NpDps5. Here, we have investigated the capacity of NpDps2 and NpDps5 to enhance stress tolerance by homologous overexpression of these two proteins in *N. punctiforme*. Both overexpression strains were found to tolerate twice as high concentrations of added H_2_O_2_ as the control strain, indicating that overexpression of either NpDps2 or NpDps5 will enhance the capacity for H_2_O_2_ tolerance. Under high light intensities, the overexpression of the two NpDps did not enhance the tolerance against general light-induced stress. However, overexpression of the heterocyst-specific NpDps5 in all cells of the filament led to a higher amount of chlorophyll-binding proteins per cell during diazotrophic growth. The OENpDps5 strain also showed an increased tolerance to ammonium-induced oxidative stress. Our results provide information of how Dps proteins may be utilised for engineering of cyanobacteria with enhanced stress tolerance.

## Teaser

The tolerance against oxidative stress targeting photosynthesis in heterocyst-forming cyanobacteria can be enhanced by homologous overexpression of specific DPS proteins.

## INTRODUCTION

Among all photosynthetic organisms, cyanobacteria are the only prokaryotes that are capable of oxygenic photosynthesis (Hamilton, Bryant and Macalady 2016). Since they produce oxygen, cyanobacteria inevitably encounter reactive oxygen species (ROS), such as hydrogen peroxide (H_2_O_2_). While ROS are important for cell signal transduction and homeostasis (Inupakutika *et al.*^2016^), they are also a natural hazard for cyanobacteria (Latifi, Ruiz and Zhang 2009). In addition, environmental stresses, such as UV radiation, high light intensities and heat, increase intracellular ROS levels dramatically (Sinha *et al.*^2002^; Hakkila *et al.*^2014^; Rastogi and Madamwar *et al.*^2015^). Uncontrolled levels of ROS will cause severe molecular damage in the cell, and cyanobacteria possess several enzymatic and non-enzymatic mechanisms involved in ROS protection (Latifi, Ruiz and Zhang 2009). A family of DNA-binding proteins from starved cells (Dps) are known to be important for hydrogen peroxide (H_2_O_2_) detoxification, and are present in most cyanobacteria (Andrews, Robinson and Rodríguez-Quiñones 2003; Latifi, Ruiz and Zhang 2009; Ekman *et al.*^2014^).

Dps proteins were first characterised from *Escherichia coli* (Almiron *et al.*^1992^), and suggested to have two modes of protecting against oxidative stress: non-specific DNA-binding (Haikarainen and Papageorgiou 2010), and ROS detoxification (Bellapadrona *et al.*^2010^). Dps proteins provide a way of sequestering intracellular iron by reversibly oxidizing ferrous iron (Fe^2+^) into ferric iron (Fe^3+^), and storing it in a mineralised iron core in the central cavity of the dodecameric protein (Haikarainen and Papageorgiou 2010). The Dps possess a conserved bimetallic active site, known as the ferroxidase center, which is analogous to that of the ferritin family and essential for this function (Alaleona *et al.*^2010^; Bellapadrona *et al.*^2010^). In contrast to ferritins and bacterioferritins (Bfr), the Dps proteins perform more efficiently when H_2_O_2_ is present as iron oxidant instead of O_2_ (Zhao *et al.*^2002^). The prevalence of harmful hydroxyl radicals, which otherwise would be generated by H_2_O_2_ and Fe^2+^ via Fenton chemistry, is thereby reduced. Dps proteins thus play an indispensable part in bacterial H_2_O_2_ scavenging and iron accumulation, simultaneously (Andrews, Robinson and Rodríguez-Quiñones 2003).

The physiological role of cyanobacterial Dps proteins has only been explored in a few studies (Shcolnick *et al.*^2009^; Ekman *et al.*^2014^; Moparthi *et al.*^2016^; Narayan *et al.*^2016^). Heterologous overexpression of cyanobacterial Dps proteins in *E. coli* resulted in enhanced protection against oxidative stress in stationary phase cultures (Nair and Finkel 2004; Castruita *et al.*^2006^; Wei *et al.*^2007^). The homologous overexpression of *all3940*, encoding a Dps in *Anabaena* sp. PCC 7120, was recently found to enhance tolerance against multiple abiotic and biotic stresses (Narayan *et al.*^2016^). The filamentous, heterocyst-forming cyanobacterium *Nostoc punctiforme* ATCC 29133 (from now on *N. punctiforme*) has five Dps proteins encoded in the genome; NpDps1 (*Npun_R3258*), NpDps2 (*Npun_F3730*), NpDps3 (*Npun_R5701*), NpDps4 (*Npun_R5799*) and NpDps5 (*Npun_F6212*) (Ekman *et al.*^2014^). *N. punctiforme* is a nitrogen-fixing cyanobacterium that can be found living independently or in symbiosis with plants and fungi. Under nitrogen limitation, 5% to 10% of the cells in a filament will differentiate into nitrogen-fixing heterocysts (Meeks *et al.*^2001^; Muro-Pastor and Hess 2012). Previous work has revealed that *N. punctiforme* shows differential expression of the five NpDps proteins when growth conditions are changed from nitrogen replete to nitrogen limiting, as well as under H_2_O_2_-induced oxidative stress (Ow *et al*. 2008, 2009; Christman, Campbell and Meeks 2011; Ekman *et al.*^2014^; Sandh, Ramström and Stensjö 2014). This expression pattern is specific to the individual NpDps proteins. The difference in the abundance of certain NpDps proteins in a H_2_ producing strain of *N. punctiforme* as compared to WT also indicates their involvement in redox regulation (Ekman *et al.*^2011^).

The NpDps1, NpDps2, NpDps3 and NpDps4 have in a phylogenetic study been characterised as Dps proteins (Ekman *et al.*^2014^). In contrast, NpDps5 clustered with a Bfr clade, mainly consisting of filamentous N_2_-fixing cyanobacteria (Ekman *et al.*^2014^). In a previous study, we demonstrated that NpDps2 is of key importance for *in vivo* H_2_O_2_ tolerance in *N. punctiforme* (Ekman *et al.*^2014^). In fact, none of the other NpDps proteins, or other reactive oxygen scavenging proteins believed to be active in the cells, could compensate for the inactivation of NpDps2 under H_2_O_2_ stress*. Npdps2* is expressed in both vegetative cells and heterocysts, but more abundantly in vegetative cells. *Npdps5*, on the other hand, is specifically expressed in heterocysts, where it is believed to primarily perform a function similar to Bfr in iron homeostasis (Ekman *et al.*^2014^). In support of this, a transcriptional study showed that the NpDps5 is co-expressed with a gene annotated as an iron permease (Moparthi *et al.*^2016^). Interestingly, although NpDps2 and NpDps5 clearly are different Dps proteins, our physiological studies of Δ*Npdps2* and Δ*Npdps5* showed that both NpDps2 and NpDps5 are necessary for maintaining fitness under elevated growth light intensity, indicating that both are essential for light induced ROS stress tolerance in *N. punctiforme* (Moparthi *et al.*^2016^), although by different mechanisms.

In this study, we continue our exploration of the roles of the typical Dps protein; NpDps2 and the atypical NpDps5, for oxidative stress tolerance. Here, we expand the repertoire of potential stress protections, by homologous overexpression of NpDps2 and NpDps5 in *N. punctiforme*. We have used the increased expression of the two NpDps proteins to determine if the tolerance to oxidative stress, induced by H_2_O_2_ and high light intensity, can be improved compared to wild type (WT) *N. punctiforme*.

## MATERIALS AND METHODS

### Bacterial strains, media and growth conditions

The filamentous cyanobacteria strain used in this study was *Nostoc punctiforme* strain ATCC 29133-S (UCD 153; Campbell, Christman and Meeks 2008). Two overexpressing mutant strains of *N. punctiforme*, OENpDps2 and OENpDps5, and a control strain, which contained an unspecific (‘empty’) plasmid were used (Table 1). Cyanobacterial cells were grown in BG11_0_ medium without nitrogen source for diazotrophic growth (Rippka *et al*. 1979), or in BG11_0_ medium with addition of NH_4_Cl (2.5 M) and 4-(2-Hydroxyethyl)piperazine-1-ethanesulfonic acid (HEPES) (5 M) for combined nitrogen growth. Seed cultures were grown at 30°C, and 45 μmol photons m^−2^ s^−1^, in 100 mL Erlenmeyer flask. Antibiotics were added (neomycin, 25 μg mL^−1^ or 12.5 μg mL^−1^ for liquid or solid medium, respectively) to all cultures harbouring the plasmid PMQAK1 (Huang *et al.*^2010^).

**Table 1.** Chl-*a*/OD_750_ ratios of control, OENpDps2 and OENpDps5 strains cultivated under NH_4_^+^-supplemented and diazotrophic growth, at two different light intensities. Chl-*a* concentration and OD_750_ were determined after four days of growth.

	NH_4_^+^ supplemented		Diazotrophic	

Photon flux (μmol m^−2^ s^−1^)	60	500	60	500
Control	3.9 ± 0.6	2.3 ± 0.3	4.7 ± 1.8	2.4 ± 0.3
OENpDps2	2.8 ± 0.7	1.6 ± 0.5	2.3 ± 0.2	2.4 ± 0.2
OENpDps5	2.3 ± 0.4	1.8 ± 0.2	3.4 ± 0.3	3.0 ± 0.4

For solid medium 1% agar (Noble agar, BD, Difco, Franklin Lakes, NJ, USA) was used. OD_750_ and Chlorophyll *a* (Chl-*a*) measurements as well as light microscopy (Axiostar, ZEISS, Jena, Germany) were performed regularly. Determinations of OD_750_ and Chl-*a* were done as in Meeks and Castenholz (1971) with modifications as follows. For the determination of Chl*-a* concentrations measured at 660 nm by using Hidex Plate CHAMELEON V (Turku, Finland) plate reader (following the manufacturer's instructions), we correlated the absorbance to the measured absorbance at 665 nm by a Varian Cary 50 (Agilent Technologies, Santa Clara, CA, USA) spectrophotometer (pathlength of 1 cm). For each sample measured by the plate reader (660 nm) we used the linear relation between the two absorbances (y = 1.6844 × – 0.0069, R^2^ = 0.9999). Biological and technical triplicates were used. Standard deviation was calculated for all biological replicates.

For nitrogen depletion of cultures, cells were washed three times in BG11_0_ medium and then resuspended in BG11_0_ to a Chl-*a* concentration of 0.5 μg mL^−1^ or stored at –80°C for protein extraction. All samples were in biological triplicates, and technical triplicates were used for all measurement. *Escherichia coli* strain DH5 *α* (Invitrogen) was used for all cloning. The *E. coli* cells were grown at 37°C in LB medium (agar or liquid) supplemented with 100 μg mL^−1^ kanamycin (Sigma-Aldrich, St. Louis, MO, USA). All strains are listed in Table S1, Supporting Information.

### Dps overexpression constructs

Two vector constructs were designed in which the genes *Npun_F3730* (*Npdps2*) and *Npun_F6212* (*Npdps5*) and the constitutive promoter P*_trc_*_2O_ (Huang *et al.*^2010^; Camsund, Heidorn and Lindblad 2014) were inserted into the shuttle vector pPMQAK1 (Huang *et al.*^2010^; Fig. S1, Supporting Information). The genes were amplified from gDNA of *N. punctiforme* using primers (Dps2_For, Dps2_Rev, Dps5_For and Dps5_Rev; Table S2, Supporting Information). P*_trc_*_2O_ was amplified from a reporter plasmid construct P*_trc_*_2O_-GFP, in pPMQAK1 (Huang *et al.*^2010^) with primers (Ptrc2o_For and Ptrc2o_Rev) in which a spacer sequence and an RBS* (TAGTGGAGGT; Heidorn *et al.*^2011^) were introduced (Table S2, Supporting Information). The DNA parts were assembled by overlap extension PCR (Bryksin and Matsumura 2010), and cloned into the vector pPMQAK1 (Huang *et al.*^2010^) using restriction digestion (EcoRI, XbaI and PstI), generating the plasmids pOEtrc2ODps2 and pOEtrc2ODps5. The empty vector pPMQAK1 in which the *ccdB* gene was removed was used as negative control (pControl). pOEtrc2ODps2, pOEtrc2ODps5 and pControl were transferred into *N. punctiforme* by conjugation (Elhai and Wolk 1988) to generate the strains OENpDps2, OENpDps5 and control, respectively.

### Protein extraction, SDS-PAGE and western blot

Cells were suspended in protein extraction buffer (50 mM Tris-HCl, 2% Triton-X, 0,4% SDS, 12.5 mM EDTA) containing a protease inhibitor cocktail (ProteaseArrest, G-Biosciences, St. Louis, MO, USA). 0.2 mL acid-washed 425–600 μm diameter glass beads (Sigma-Aldrich) were mixed with the cells. Cells were disrupted using the Precellys-24 homogeniser (Bertin Instruments, Montigny-le-Bretonneux, France) during 4 × 30 s. Centrifugation was done twice at 18 000 *× g*, for 10 min at 4°C. Protein concentration was determined by using the DC protein assay (Bio-Rad, Hercules, CA, USA). Ten micrograms of proteins, per well, were separated by sodium dodecyl sulfate–polyacrylamide gel electrophoresis (SDS-PAGE), using Any kD gels (Bio-Rad), and transferred to 0.2 μm Polyvinylidene fluoride (PVDF) membrane using the Trans-Blot Turbo Transfer System (Bio-Rad). Strep-tags were detected by Anti-Strep-tag ΙΙ (Abcam, Cambridge, UK) by Clarity Western ECL substrate (Bio-Rad) using standard techniques.

### Hydrogen peroxide and light treatments

All measurements were performed the same way for diazotrophic and ammonium (NH_4_^+^) supplemented cultures. For the H_2_O_2_ experiments, the control, OENpDps2 and OENpDps5 strains were cultivated in 6-well plates (8 mL culture per well) with 120 rpm continuous shaking for four days at 30°C, under 45 μmol photons m^−2^ s^−1^, with a starting Chl-*a* concentration of 0.5 μg mL^−1^. Liquid H_2_O_2_ was added to final concentrations of 0, 0.5, 1.0, 1.5, 2.5, 3.5 and 5.0 mM, at start and at day three of cultivation. For the light experiments, cultures were grown as described above, but under 60 and 500 μmol photons m^−2^ s^−1^. The Heliospectra LX60 (Gothenburg, Sweden) system with controlled 5700K LED white light was used for the 500 μmol photons m^−2^ s^−1^ illumination. All experiments were performed on at least three independent biological replicates.

### Oxygen evolution measurements

For oxygen evolution measurements, cultures of the control, the OENpDps2 and OENpDps5 strains were assayed after 4 days of cultivation in 50 mL Erlenmeyer flask under 60 and 500 μmol photons m^−2^ s^−1^, in three biological replicates each. A few minutes prior to measurement, the cultures were transferred from the specified growth conditions, and placed in the measuring chamber. The cell suspension was diluted in BG11_0_ medium to a Chl-*a* concentration at approximately 1 μg Chl-*a* mL^−1^. The O_2_ evolution was assayed at 25°C using a Clark-type oxygen electrode (Hansatech, King's Lynn, UK), in three biological and three technical replicates for each strain. Saturating illumination was provided with a 150 W slide projector lamp, equipped with a bandpass interference filter with transmittance between 520 and 630 nm (Schott Glass Technologies Inc., Mainz, Germany).

## RESULTS

### Homologous overexpression of NpDps2 and NpDps5

Two overexpression strains, OENpDps2 and OENpDps5, were constructed from *Nostoc punctiforme* by insertion of the genes *Npun_3730* (*Npdps2*) and *Npun_6212 (Npdps5)* into the vector pPMQAK1 downstream of the constitutive promoter Ptrc2O (Fig. S1, Supporting Information; Huang *et al.*^2010^). The non-native Ptrc2O promoter is reported as a strong constitutive promoter without cell specificity in *N. punctiforme* (Camsund, Heidorn and Lindblad 2014). The NpDps proteins were tagged with a C-terminal Strep(II)-tag as a fusion protein, for detection by western blotting. As a phenotype control, a strain was constructed from *N. punctiforme* containing the pPMQAK1 plasmid without the genes encoding the NpDps proteins and Strep-tag (Fig. S1, Supporting Information).

**Figure 1.**
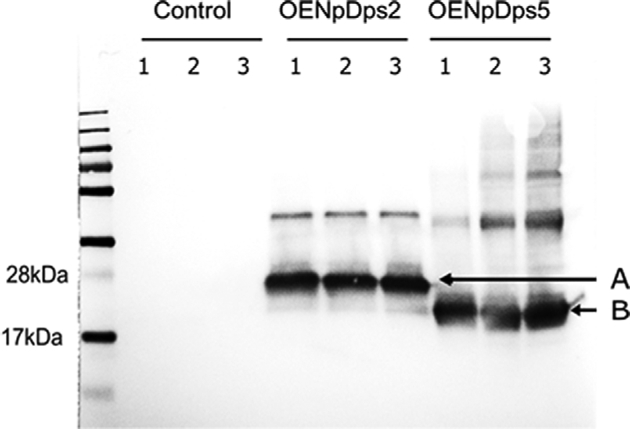
Immunoblot of Strep(II)-tagged NpDps proteins. Total protein extracts of strains; control, OENpDps2 and OENpDps5 were analysed using chemiluminescent detection of the Strep(II)-tag, showing the presence of NpDps2-strep(II)tag and NpDps5-strep(II)tag fusion proteins from cells cultured at different light intensities. From left to right: MW ladder (PageRuler); in sets of three lanes—control strain, the OENpDps2 strain and the OENpDps5 strain. Three samples from each strain were analysed: before treatment (1), after 5 days under 60 μmol photons m^−2^ s^−1^ (2), and after 5 days under 500 μmol photons m^−2^ s^−1^ (3). The bands at 20 kDa (A) and 18 kDa (B) indicate the NpDps2-strep(II)tag fusion protein and NpDps5-strep(II)tag fusion protein, respectively.

Prior to protein extraction and western blotting, the control, OENpDps2 and OENpDps5 strains were cultivated for 4 days under 60 μmol photons m^−2^ s^−1^, and 500 μmol photons m^−2^ s^−1^. Using antibodies against the Strep(II)-tag, bands at ∼20 kDa, corresponding to NpDps2-Tag, and ∼18 kDa for the NpDps5-Tag fusion proteins could be detected (Fig. 1). This clearly shows that the target NpDps proteins were expressed as Strep(II)-tagged fusion proteins with correct sizes. As expected, the control strain containing the empty pPMQAK1 vector did not produce any signal. The western blot suggests that the expression levels of the Strep-tagged NpDps2 and NpDps5 were rather unaffected by the different light regimes. The increased levels of NpDps2 and NpDps5 transcripts in the overexpression strains as compared to the endogenous transcript level of these two Dps proteins were confirmed by RT-qPCR (Fig. S2, Supporting Information).

### Tolerance to H_2_O_2_ stress in Dps overexpression strains

To test the tolerance to H_2_O_2_ in the overexpression strains, varying concentrations of H_2_O_2_ were added during growth in liquid cultures. The control strain, and the OENpDps2 and OENpDps5 strains, were grown for 4 days, either diazotrophically or supplemented with combined nitrogen in the form of ammonium salt (NH_4_^+^). Additions of liquid H_2_O_2_ were made to the cultures at two occasions during the experiment; at the start (day zero), and after 3 days of cultivation.

**Figure 2.**
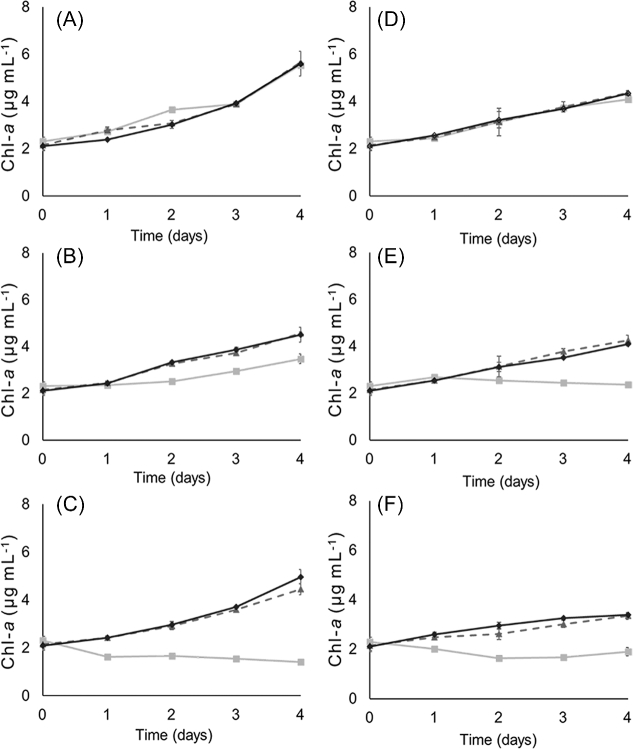
Growth as determined by Chl-*a* concentration in cultures of *N. punctiforme* strains under addition of different amounts of H_2_O_2_. Diazotrophic cultures (**A**–**C**) and NH_4_^+^-supplemented cultures (**D**–**F**) of *N. punctiforme* strains control (squares, light grey line), OENpDps2 (triangles, dashed grey line) and OENpDps5 (diamonds, black line) strains. Additions of H_2_O_2_ were made to final concentrations to 0.5 mM (A and D), 1.5 mM (B and E), and 3.5 mM (C and F). Each sample was measured in biological and technical triplicates and the error bars indicate standard deviation of the sample.

The growth, as determined by Chl-*a* concentration, indicates that both overexpression strains tolerate additions of up to 3.5 mM H_2_O_2_ under both diazotrophic and NH_4_^+^-supplemented growth. While the addition of 3.5 mM H_2_O_2_ led to a slower increase in growth, it did not stop growth completely (Fig. 2). In contrast, the control strain showed decreased growth already after addition of 1.5 mM H_2_O_2_, and died after addition of 3.5 mM H_2_O_2_ to diazotrophic culture (Fig. 2B and C). Moreover, when grown in NH_4_^+^-supplemented medium, the control strain displayed severe growth inhibition already by addition of 1.5 mM H_2_O_2_ (Fig. 2E). This result shows that overexpression of the NpDps2 and NpDps5 increases tolerance to oxidative stress from H_2_O_2_.

Interestingly, when grown diazotrophically the two overexpression strains displayed different degrees of tolerance to high amounts of H_2_O_2_. The OENpDps5 strain showed a capacity to continue to grow even after addition of 5.0 mM of H_2_O_2_ under diazotrophic growth (Fig. S3B, Supporting Information). The OENpDps2 strain on the other hand was severely impaired from the addition of 5.0 mM H_2_O_2_ under diazotrophic growth. In NH_4_^+^-supplemented cultures, both overexpression strains survived but did not show any growth after addition of 5 mM H_2_O_2_ (Fig. S3A, Supporting Information).

### Tolerance to light stress in Dps overexpression strains

Photosynthetic organisms are constantly at risk of light energy-induced stress. In a previous study, where we studied the knock-out strains Δ*Npdps2* and Δ*Npdps5*, we demonstrated that NpDps2 and NpDps5 are involved in acclimation to high light intensities in *N. punctiforme* (Moparthi *et al.*^2016^). To investigate if an increased abundance of the NpDps proteins could enhance the tolerance to high light stress, we analysed the OENpDps2 and OENpDps5 strains after cultivation at moderate light intensity (60 μmol photons m^−2^ s^−1^), and high light intensity (500 μmol photons m^−2^ s^−1^).

**Figure 3.**
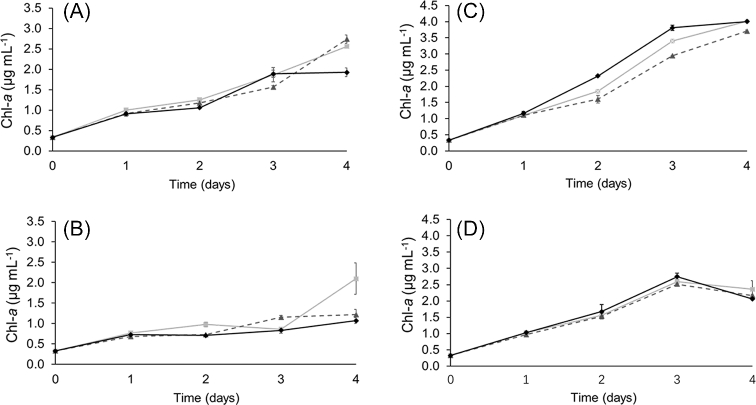
Growth as determined by Chl-*a* concentration in cultures of *N. punctiforme* strains under different light intensities. Diazotrophic cultures (**A** and **B**) and NH_4_^+^-supplemented cultures (**C** and **D**) for *N. punctiforme* strains control (squares, light grey line), OENpDps2 (triangles, dashed grey line) and OENpDps5 (diamonds, black line). Strains were grown at 60 μmol photons m^−2^ s^−1^ (A and C) and 500 μmol photons m^−2^ s^−1^ (B and D). Each sample was measured in biological and technical triplicates and the error bars indicate standard deviation of the sample.

Fig. 3 shows the growth, measured as Chl-*a* concentration, of all strains under diazotrophic conditions (Fig. 3A and B) and NH_4_^+^-supplementation (Fig. 3C and D). At 60 μmol photons m^−2^ s^−1^, all three strains grew to a similar Chl-*a* concentration at day four, in both diazotrophic and NH_4_^+^-supplemented cultures (Fig. 3A and C). In contrast, at 500 μmol photons m^−2^ s^−1^ and diazotrophic growth, the overexpression strains had less total Chl-*a* than the control strain after 4 days (Fig. 3B). The cell density was measured in all cultures by both Chl-*a* concentration and by OD at 750 nm. The Chl-*a*/OD_750_ was not different in overexpression strains grown diazotrophically at 500 μmol photons m^−2^ s^−1^, compared to the same strains grown in 60 μmol photons m^−2^ s^−1^ (Table 1). This means that the Chl-*a* per cell did not vary in the overexpression strains grown under diazotrophy. We therefore conclude that overexpression of NpDps proteins impedes diazotrophic growth at 500 μmol photons m m^−2^ s^−1^, compared to the control strain where Dps proteins are expressed to a normal degree. It could be that overexpression takes a toll on all cellular processes under the combined stresses of high light and the need of N_2_ fixation. Under NH_4_^+^-supplemented growth, all three strains had reduced Chl-*a* content at day four of high light treatment (Fig. 3D).

From the growth at different light intensities suggest that overexpression of NpDps2 and NpDps5 did not enhance the light-induced stress tolerance of *N. punctiforme*. However, the capacity for coping with light stress does not only affect the growth rate. Light-induced stress management in cyanobacteria typically involves the fitness of the photosynthetic apparatus (Sonoike *et al.*^1997^; Nishiyama *et al.*^2001^; Murata *et al.*^2007^). To investigate the photosynthetic fitness of the overexpression strains we measured the O_2_ evolution activity in the cultures after four days of growth.

**Figure 4.**
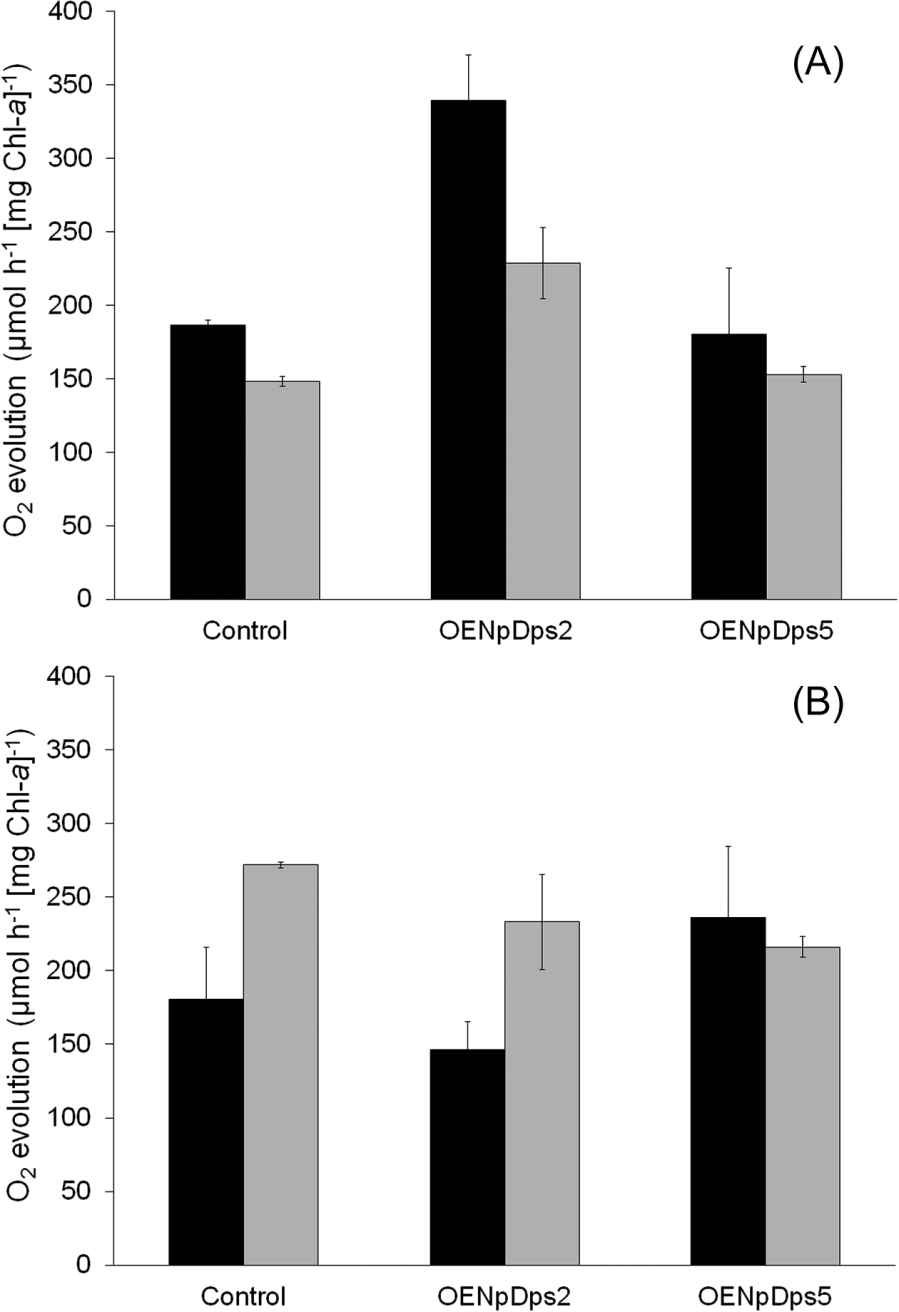
Oxygen evolution. NH_4_^+^-supplemented (black bars) or diazotrophic (grey bars) cultures of the *N. punctiforme* strains control, OENpDps2 and OENpDps5. The strains were grown at 60 μmol photons m^−2^ s^−1^ (**A**) and 500 μmol photons m^−2^ s^−1^ (**B**). Samples were taken from each strain after 4 days of growth as in Fig. 4 and measured with a Clark electrode. Each sample was measured in biological and technical triplicates and the error bars denote corrected sample standard deviations.

At 60 μmol photons m^−2^ s^−1^, the O_2_ evolution per mg Chl-*a* was generally higher in the NH_4_^+^-supplemented cultures than in diazotrophic cultures on a Chl-*a* basis (Fig. 4A). However, O_2_ evolution per cell at the two culture conditions was the same, when the Chl-*a*/OD ratio is compensated for (Table 1). At 60 μmol photons m^−2^ s^−1^, the control and OENpDps5 strains, respectively, had very similar O_2_ evolution activities under both NH_4_^+^-supplemented (Fig. 4A; black bars) and diazotrophic growth (Fig. 4A; grey bars). In contrast, the OENpDps2 strain showed up to twice as high O_2_ evolution activity than the other two strains, under both nutrient growth conditions (Fig. 4A). By comparison of the Chl-*a* content and the OD_750_, we could conclude that the OENpDps2 strain had a lower number of photosynthetic components per cell than the control (Table 1; Table S5, Supporting Information). The higher O_2_ evolution activity in the OENpDps2 strain at 60 μmol photons m^−2^ s^−1^ could be a sign that the photosynthetic activity is higher in this strain, either at the level of photosynthetic electron transfer or downstream at the level of carbon fixation.

At 500 μmol photons m^−2^ s^−1^, the OENpDps2 strain had slightly decreased O_2_ evolution activity compared to the control strain under both nutrient growth conditions (Fig. 4B), indicating that the photosynthetic activity in OENpDps2 was more sensitive to high-light treatment than in the control strain. This result corroborates the growth results, and indicates that overexpression of NpDps2 made the strain less tolerant to light stress. On the other hand, both the control and the OENpDps2 strains had clearly lower O_2_ evolution activity in the NH_4_^+^-supplemented cultures at 500 μmol photons m^−2^ s^−1^, (Fig. 4B; black bars), compared to the diazotrophic cultures (Fig. 4B; grey bars). The deterioration of O_2_ evolution activity at NH_4_^+^-supplementation may be due to toxicity of NH_4_^+^, which is particularly damaging to Photosystem II (Zhu *et al.*^2000^; Drath *et al.*^2008^).

However, the OENpDps5 strain had virtually the same O_2_ evolution activity under both nutrient conditions at 500 μmol photons m^−2^ s^−1^. Interestingly, during NH_4_^+^-supplemented growth, the O_2_ evolution activity in the OENpDps5 strain was higher than in the other two strains at this light intensity, and very similar to the O_2_ evolution activity of OENpDps5 under diazotrophic growth (Fig. 4B). Thus, it seems that the OENpDps5 strain did not suffer from the presence of NH_4_^+^ during high-light conditions as much as the other two strains did.

## DISCUSSION

We have previously observed that stress tolerance in *N. punctiforme* is considerably weakened in the deletion strains Δ*Npdps2* and Δ*Npdps5* (Ekman *et al.*^2014^; Moparthi *et al.*^2016^). Our physiological studies showed that, although NpDps2 and NpDps5 clearly are different Dps proteins with individual roles, both these proteins are necessary for maintaining cellular fitness. We found that NpDps2 is of particular importance for combatting oxidative stress induced by H_2_O_2_. In addition, both NpDps2 and NpDps5 are involved in tolerance to high light intensities, indicating that these NpDps are essential for light-induced ROS stress tolerance in *Nostoc punctiforme* (Moparthi *et al.*^2016^) by different mechanisms. However, the ultimate goal with our work is to elucidate to what extent stress tolerance is of importance in the design of robust production strains for biotechnological purposes. The two overexpression strains OENpDps2 and OENpDps5 were therefore constructed to test if these could enhance cell fitness beyond current levels.

Both the OENpDps2 and OENpDps5 strains were indeed found to have an increased endurance towards oxidative stress. The overexpression strains tolerated more than twice as much added H_2_O_2_ as the control strain, under both diazotrophic and ammonium-supplemented growth conditions (Fig. 2, Fig. S3, Supporting Information). The NpDps2 has been indicated as a ‘classic’ Dps protein involved in H_2_O_2_ detoxification, while NpDps5 is heterocyst specific and hypothesised to function in iron storage and regulation (Ekman *et al.*^2014^). Interestingly, and uniquely for this study, the results from overexpressing NpDps5 in the entire filament, indicate that it also is capable of providing its host with enhanced H_2_O_2_ tolerance. This was especially pronounced under diazotrophic growth, where the OENpDps5 strain was able to withstand higher concentrations of H_2_O_2_ for a prolonged time period than the OENpDps2 strain. This reinforces our previous conclusion that NpDps5 plays a critical role in oxidative stress control under N_2_-fixation (Moparthi *et al.*^2016^). The increased H_2_O_2_ tolerance might be an effect of enhanced uptake of ferrous iron, and thereby a reduction in the production of toxic hydroxyl radicals by Fenton chemistry. This is an important conclusion that is likely to have an impact on future design of engineered cyanobacterial strains.

Cyanobacteria are sensitive to high light intensities, as this increases the pressure from oxidative stress (Sonoike *et al.*^1997^; Nishiyama *et al.*^2001^; Murata *et al.*^2007^). Many isolated strains are therefore grown at moderate light intensities of 20–50 μmol photons m^−2^ s^−1^ (Islam and Beardall 2017). When cultivated at 60 μmol photons m^−2^ s^−1^, the control strain had a considerably higher concentration of Chl-*a* per OD at 750 nm (Table 1) than both overexpression strains, indicating that more photosynthetic proteins were present in the cells of the control strain. Interestingly, however, at 60 μmol photons m^−2^ s^−1^, the O_2_ evolution per Chl-*a* was twice as high in the OENpDps2 strain as in the control strain under NH_4_^+^-supplemented growth, and 1.5 times higher under diazotrophic growth (Fig. 4A). Translated to O_2_ evolution per cell, this activity was similar in the control and OENpDps2 strains (Table 1). This may be an indication that the OENpDps2 strain is capable of compensating for a lesser amount of Chl-*a* per OD_750_, by using the photosynthetic capacity more efficiently. A more thorough investigation would be needed to fully understand the effect on the photosynthetic efficiency.

Neither of the overexpression strains grew better than the control at high light intensity. However, during diazotrophic growth at 500 m^−2^ s^−1^, the OENpDps5 strain had higher amounts of Chl-*a* per OD_750_ than in both the other strains. This result underscores the importance of NpDps5 for nitrogen fixation, and for the stability of photosynthetic proteins (Ekman *et al.*^2014^; Moparthi *et al.*^2016^). It is known that heterocysts have higher abundance of Photosystem I than vegetative cells in *N. punctiforme* (Ow *et al.*^2009^). It is thus possible that NpDps5 might be of assistance for Photosystem I assembly or activity in diazotrophic cultures.

Another possibility is that overexpression of NpDps5 provides a more general protection to both photosystems. This was supported by the O_2_ evolution activity in the NH_4_^+^-supplemented cultures: the control and OENpDps2 strains had lower O_2_ evolution activities at 500 μmol photons m^−2^ s^−1^ and thus seemed to suffer more from high-light stress in the NH_4_^+^-supplemented cultures than during diazotrophic growth. Interestingly, the O_2_ evolution activities in the OENpDps5 strain were similar for the two nutrient conditions at high light intensity, indicating that the OENpDps5 strain was unharmed by NH_4_^+^ (Fig. 4B). It has been suggested that the presence of ammonia at high light intensities can be inhibiting for Photosystem II, and may involve formation of ROS (Zhu *et al.*^2000^). Although the exact mechanism is unknown, our results indicate that the harmful effect of NH_4_^+^ may be counteracted by NpDps5, and suggest that NpDps5 has a protective effect on the photosynthetic apparatus against oxidative stressors. This protective role of NpDps5 is normally localised to heterocysts but may be extended to vegetative cells via overexpression.

To conclude, in the present study, we have observed that an increased stress tolerance in *N. punctiforme* can be obtained by overexpression of NpDps2 and NpDps5. We suggest that the Bfr-like protein NpDps5 is able to enhance the capacity of producing photosynthetic proteins under high light stress in diazotrophic cultures. Under our experimental conditions the overexpression itself was to some extent harmful when it came to stress management under high light intensities. However, this should be possible to overcome by fine-tuning the expression levels of the NpDps, and thus be used to increase the high light tolerance in *N. punctiforme*.

## SUPPLEMENTARY DATA

Supplementary data are available at *FEMSLE* online.

## Supplementary Material

Supplementary Data
