# Supplementary material for: Homologous overexpression of NpDps2 and NpDps5 increases the tolerance for oxidative stress in the multicellular cyanobacterium Nostoc punctiforme
Source: FEMS Microbiol Lett. 2018 Aug 13;365(18):fny198. doi: 10.1093/femsle/fny198 (PMC6116882; doi:10.1093/femsle/fny198)
Supplement: Supplementary Data [file fny198_supplemental_files.docx]

**Supporting information**

Homologous overexpression of NpDps2 and NpDps5 increases the tolerance for oxidative stress in the multicellular cyanobacterium *Nostoc punctiforme*.

Authors: Xin Li; Henna Mustila; Ann Magnuson; Karin Stensjö*.

Department of Chemistry – Ångström Laboratory, Uppsala University, SE 75120 Uppsala, Sweden

*Corresponding author. Email address: karin.stensjo@kemi.uu.se; Phone: +46 (0)18 471 6586; Fax: +46 (0)18 471 6844

Additional Supporting Information may be found in the online version of this article

**Table S1.** Bacterial strains and plasmids used in this work.

**Table S2.** Primers used in this work.

**Table S3.** Chlorophyll-*a*/OD_750_ ratios of cells, under NH_4_^+^ supplemented and diazotrophic growth, treated with different H_2_O_2_ concentrations.

**Table S4**. OD_750_ of cells, under NH_4_^+^ supplemented and diazotrophic growth, treated with different H_2_O_2_ concentrations.

**Table S5**. OD_750_ of cells, under NH_4_^+^ supplemented and diazotrophic growth, treated with different light intensities.

**Fig. S1.** Overview of Dps overexpression vector constructs.

**Fig. S2.** Transcript abundance levels of *Npdps2* and *Npdps5* in the control strain and in the overexpression strains, OE-NpDps2 and OE-NpDps5, analyzed with RT-qPCR.

**Fig. S2.** Materials and methods.

**Fig. S3.** Growth of *Nostoc punctiforme* strains: OENpDps5, OENpDps2, and Control following treatment with 5.0 mM H_2_O_2_.

| **Table S1.** Bacterial strains and plasmids used in this work. | |  |
| --- | --- | --- |
| Bacterial strain and plasmid | Genotype and information | Reference/source |
| *Escherichia coli* strain DH5α |  | (Taylor et al., 1993) |
| *Nostoc punctiforme* ATCC 29133-S | UCD 153, natural mutant of WT *N. punctiforme* | (Campbell et al., 2008) |
| OENpDps2 | *N. punctiforme* with NpDps2 (encoded by *Npun_F3730*) overexpression construct | This study |
| OENpDps5 | *N. punctiforme* with NpDps5 (encoded by *Npun_F6212*) overexpression construct | This study |
| Control | *N. punctiforme* with pPMQAK1 | This study |
| Plasmid pPMQAK1 | Km^r^, Amp^r^, P*_trc2O_* promoter | (Camsund et al. , 2014; Huang et al., 2010) |

**Table S2.** Primers used in this work. The Strep(II)-tag and stop codon are indicated by underline and dotted underline respectively. The RBS* sequence is indicated by double underline.

| **Primers** | **Sequence 5’-3’** |
| --- | --- |
| Ptrc2o_For | GCAGAATTCAAATGTGAGCGAGTAACAACCTG |
| Ptrc2o_Rev | CATTCTAGAACCTCCACTAGGTCAATGTGTGAAATTGTGAGCGCTCACAA |
| Dps2_For | TTGACCTAGTGGAGGTTCTAGAATGTCATCAAAAGTAACAGTCA |
| Dps2_Rev | GCGCTGCAGCGGCCGCTACTAGTTGTACATTATTATTTTTCAAATTGGGGATGACTCCACTATTCTTCCAGTAAACTTCTCAACA |
| Dps5_For | TTGACCTAGTGGAGGTTCTAGAATGCAAGAACTTGACTATAA |
| Dps5_Rev | GCGCTGCAGCGGCCGCTACTAGTTGTACATTATTATTTTTCAAATTGGGGATGACTCCATTAGCTAAAATCGCGTAGCATC |

**Table S3.** Chlorophyll-*a*/OD_750_ ratios of cells cultivated under NH_4_^+^ supplemented and diazotrophic growth treated with H_2_O_2_ concentrations from 0 to 5.0 mM cells were grown in 6-well plates. Chlorophyll-*a* concentration and OD_750_ were measured after four days of H_2_O_2_ treatment.

|  | NH_4_^+^ supplemented | | | Diazotrophic | | |
| --- | --- | --- | --- | --- | --- | --- |
| H_2_O_2_ (mM) | 0 | 3.5 | 5.0 | 0 | 3.5 | 5.0 |
| Control | 10.1±1.1 | 5.9±0.4 | 2.9±0.2 | 8.7±0.8 | 3.9±0.3 | 3.2±0.1 |
| OENpDps2 | 10.5±0.3 | 8.5±0.8 | 6.4±1.3 | 9.2±1.2 | 8.6±1.0 | 5.0±1.0 |
| OENpDps5 | 10.2±0.3 | 8.1±0.5 | 7.1±0.7 | 10.0±1.1 | 9.5±0.8 | 7.5±0.3 |

**Table S4.** OD_750_ values for the Control, OENpDps2 and OENpDps5 strains under NH_4_^+^ supplemented and diazotrophic growth, either without addition of H_2_O_2_ or with addition of 3.5 mM. Cells were grown in 6-well plates. OD_750_ was measured after four days of H_2_O_2_ treatment.

|  | NH_4_^+^ supplemented | | Diazotrophic | |
| --- | --- | --- | --- | --- |
| H_2_O_2_ (mM) | 0 | 3.5 | 0 | 3.5 |
| Control | 0.73±0.28 | 0.53±0.20 | 1.1±0.30 | 0.59±0.09 |
| OENpDps2 | 0.73±0.43 | 0.65±0.16 | 1.1±0.18 | 0.87±0.27 |
| OENpDps5 | 0.72±0.24 | 0.69±0.08 | 1.1±0.43 | 0.88±0.35 |

**Table S5**: OD_750_ values for the control, OENpDps2 and OENpDps5 strains in samples used for oxygen evolution measurements, after 4 days of growth at two different light intensities.

|  | NH_4_^+^ supplemented | | | Diazotrophic | |  |
| --- | --- | --- | --- | --- | --- | --- |
| Photon flux (µmol m^-2^ s^-1^) | 60 | 500 | 60 | | 500 | |
| Control | 0.32±0.03 | 0.26±0.03 | 0.37±0.04 | | 0.37±0.02 | |
| OENpDps2 | 0.28±0.05 | 0.41±0.05 | 0.26±0.03 | | 0.44±0.07 | |
| OENpDps5 | 0.29±0.01 | 0.32±0.07 | 0.31±0.02 | | 0.47±0.14 | |

**Fig. S1**


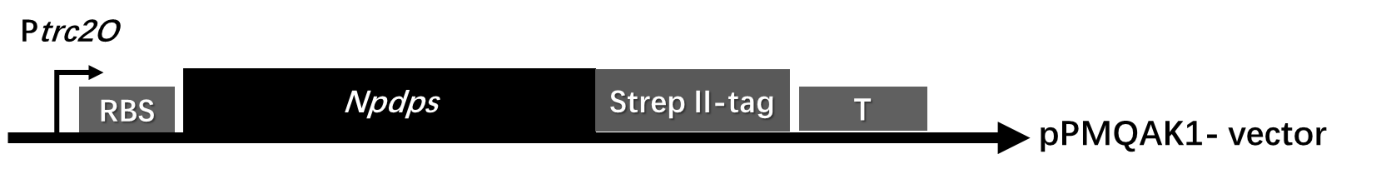


**Fig. S1.** Overview of Dps overexpression vector constructs. The genes encoding the two Dps proteins NpDps2 and NpDps5 were inserted into the self-replicating vector pPMQAK1 downstream of the constitutive promoter Ptrc2O. A C-terminal Strep(II)-tag was fused to the Dps.

**Fig. S2**

**
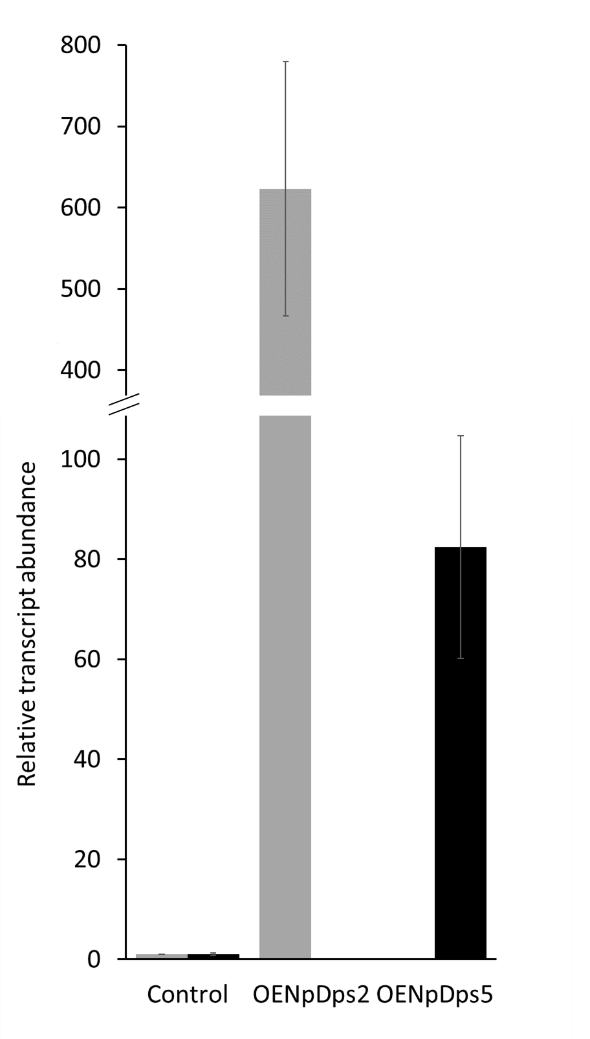
**

**Fig. S2.** Transcript abundance levels of *Npdps2* (grey bars) and *Npdps5* (black bars) in the control strain and in the overexpression strains, OE-NpDps2 and OE-NpDps5, analyzed with RT-qPCR. RNA was isolated from N_2_-fixing cultures grown under 60 µmol photons m^−2^ s^−1^ at 30 °C. The values are the mean of three biological replicates and error bars indicate standard deviation. Data are presented as fold change normalized to the expression level of control strain.

**Fig S2. Material and Methods**

For total RNA isolation, N_2_-fixing cultures were harvested by centrifugation at 1000 x g, for 5 min, at 4°C. RNA was extracted with TRI reagent (Sigma-Aldrich) following the protocol in Agervald et al. (2008). Contamination of DNA was avoided by using TURBO DNA-free Kit (Thermo Fisher Scientiﬁc) according to manufacturer’s instructions. Synthesis of cDNA was performed using the iScript™ Reverse Transcription Supermix for RT-qPCR (Bio-Rad) according to the manufacturer's protocol. RNA samples with no added reverse transcriptase as well as a sample without a template were used as a negative control in RT-qPCR run. RT-qPCR was performed using PerfeCTa SYBR Green SuperMix (Quanta BioSciences) according to the manufacturer's instructions, on a CFX Connect Real-Time PCR Detection System (Bio-Rad). Gene specific primers described in Moparthi et al. (2016) were used for *Npdps2* and *Npdps5*, and *rnpB* was used as reference gene. The changes in mRNA levels relative to the control were calculated using comparative Ct method as described in Livak et al. (2001).

**References for** **Fig S2. Material and Methods**

Agervald Å, Stensjö K, Holmqvist M, Lindblad P. Transcription of the extended hyp-operon in *Nostoc* sp. strain PCC 7120, BMC Microbiol. 2008; 8:69.

Livak KJ, Schmittgen TD. Analysis of relative gene expression data using real-time quantitative PCR and the 2(-Delta Delta C(T)) Method. Methods. 2001; 25:402-8.

Moparthi V, Li X, Vavitsas K, Dzhygyr I, Sandh G, Magnuson A, Stensjö K. The two Dps proteins, NpDps2 and NpDps5, are involved in light-induced oxidative stress tolerance in the N_2_-fixing cyanobacterium *Nostoc punctiforme*. Biochimica et Biophysica Acta – Bioenergetics 2016; 1857:1766-1776

**Fig. S3**


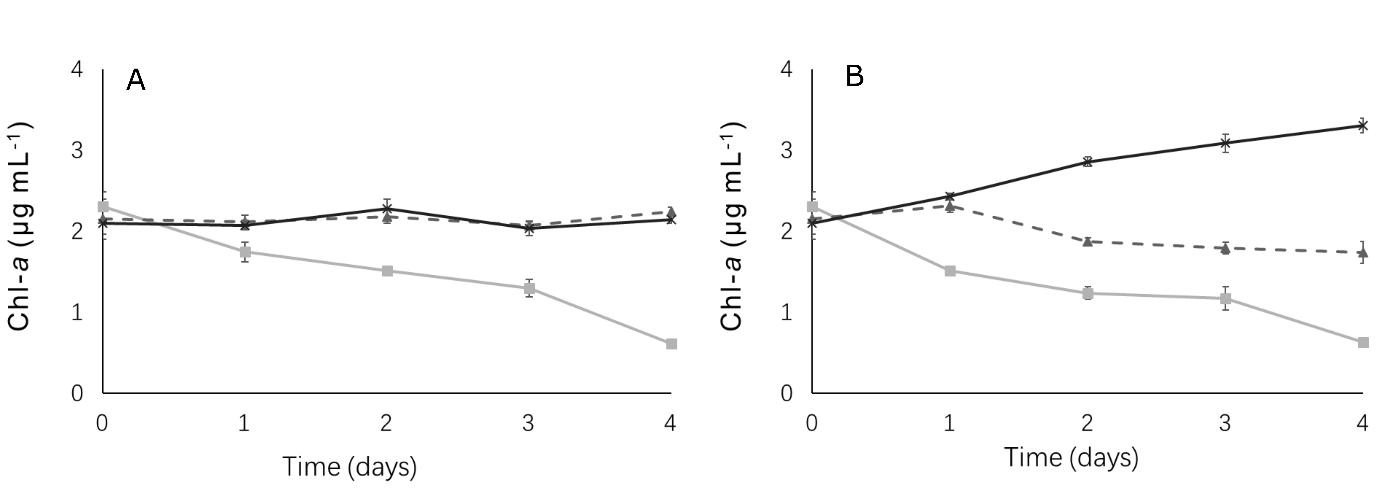


**Fig. S3. Growth under 5 mM H_2_O_2_.** *Nostoc punctiforme* strains, Control (squares and unbroken, light grey line), OENpDps2 (triangles and dashed grey line) and OENpDps5 (crosses and black line) strains under NH_4_^+^-supplemented (A) and diazotrophic (B) growth with addition of 5.0 mM of H_2_O_2_. The growth was determined by Chlorophyll-*a* concentration per mL of culture. Each sample was measured in biological and technical triplicates and the error bars indicate standard deviation of the sample.
